# Analysis of Aroma Components in 30 Varieties of Peony Flowers

**DOI:** 10.3390/plants15213490

**Published:** 2026-11-08

**Authors:** Fang Xu, Keer Wang, Yi Li, Peng Zhang

**Affiliations:** 1 Public Analytical and Testing Center, Beijing Forestry University, Beijing 100083, China; 2 School of Landscape Architecture, Beijing Forestry University, Beijing 100083, China; aesculus_chinensis@icloud.com (K.W.); 13951965552@163.com (Y.L.); gzyyndemailbox@163.com (P.Z.)

**Keywords:** *Paeonia lactiflora*, floral volatiles, ROAV, PCA, dynamic headspace sampling

## Abstract

In this study, dynamic headspace sampling combined with automated thermal desorption–gas chromatography/mass spectrometry (ATD–GC/MS) was employed to profile the volatile emissions from fresh flowers of 30 Chinese herbaceous peony cultivars (*Paeonia lactiflora* Lactiflora Group). Relative odor activity values (ROAV) were calculated to pinpoint key aroma-active constituents, and principal component analysis (PCA) was applied to examine chemometric relationships among the cultivars. The volatile blends were predominantly composed of terpenoids, benzenoids, and alcohols. Ten compounds—namely ocimene, β-terpinene, terpinolene, 1,4-dimethoxybenzene, phenethyl alcohol, 1-pentanol, eucalyptol, linalool, citronellol, and cis-3-hexenyl acetate—exhibited ROAV > 10 and were thus recognized as major odor contributors, jointly conferring rosy, citrusy, sweet, woody, and herbaceous–green nuances. Cultivar-to-cultivar variations in the dominant odorants, such as ocimene in ‘Jinzanciyu’, citronellol in ‘Chifen’, and linalool in ‘Dongfanghong’, gave rise to distinctive sensory signatures. Furthermore, PCA score plots revealed a clustering trend among cultivars with similar petal colors, suggesting a possible linkage between floral pigmentation and volatile composition. Collectively, these results offer a chemical foundation for fragrance typology and serve as a useful reference for scent-oriented breeding programs in herbaceous peonies.

## 1. Introduction

*Paeonia lactiflora* is an herbaceous perennial that boasts a lengthy cultivation history in China and ranks among the country’s most cherished traditional ornamentals. Historical records indicate that it was one of the earliest florally named plants in Chinese horticulture, as captured in the adage that “among all the flowers, its name is the oldest” [1]. The ornamental appeal of peony blooms lies in their substantial size, multilayered petal arrangements, and diverse flower forms. Beyond aesthetic value, this species also offers notable edible and medicinal benefits, having long been integrated into traditional culture and horticultural practice [2].

Floral fragrance arises from low-molecular-weight volatile secondary metabolites emitted by blossoms [3]. These volatiles serve key ecological functions, including pollinator attraction and defense against herbivores and pathogens [4,5]. They also find wide-ranging uses in the perfume, cosmetic, and pharmaceutical sectors [6,7]. As a critical ornamental attribute, floral scent is largely dictated by the qualitative and quantitative makeup of emitted volatiles [8,9]. Prior investigations have demonstrated that scent profiles differ substantially across plant species and even among conspecific cultivars, owing to underlying genetic variability [10,11,12]. Marked variation in volatile composition has likewise been documented among *P. lactiflora* cultivars. Existing evidence points to terpenes and alcohols as the predominant components of peony fragrance, with esters, aldehydes, ketones, and ethers typically occurring at much lower abundances [13]. Nevertheless, much of the current work has concentrated on interspecific or intercultivar volatile comparisons [14]. Consequently, a definitive aroma signature for peony has yet to emerge, and standardized fragrance-type classification criteria remain absent.

The volatile profile of flowers is shaped not only by genotypic differences but also by the choice of sampling and analytical protocols [15]. Common approaches for floral scent characterization include headspace sampling, solid-phase microextraction (SPME), and gas chromatography–mass spectrometry (GC–MS) [16]. However, collecting detached floral tissues can induce physiological stress, potentially altering volatile biosynthesis and release [17]. By contrast, dynamic headspace collection—which draws volatiles directly from intact plants via an air pump onto sorbent materials [18,19]—coupled with automated thermal desorption–gas chromatography/mass spectrometry (ATD–GC–MS) offers a straightforward, highly reproducible, solvent-free alternative. This approach minimizes physical disturbance to plant tissues [20] and thus yields a more faithful representation of volatile emission under natural growing conditions [21,22].

In the present work, we selected ornamental cultivars from the Chinese *P. lactiflora* cultivar group to characterize the volatile components of fresh flowers. We determined the composition and relative abundances of aroma compounds, evaluated cultivar-related differences in fragrance intensity, pinpointed key odor-active constituents, and applied PCA to delineate scent patterns across cultivars. The outcomes of this study are expected to deepen our understanding of fragrance traits in Chinese peony cultivars and to furnish a practical reference for the evaluation and taxonomic classification of peony floral scents.

## 2. Materials and Methods

### 2.1. Plant Materials

**Figure 1:**
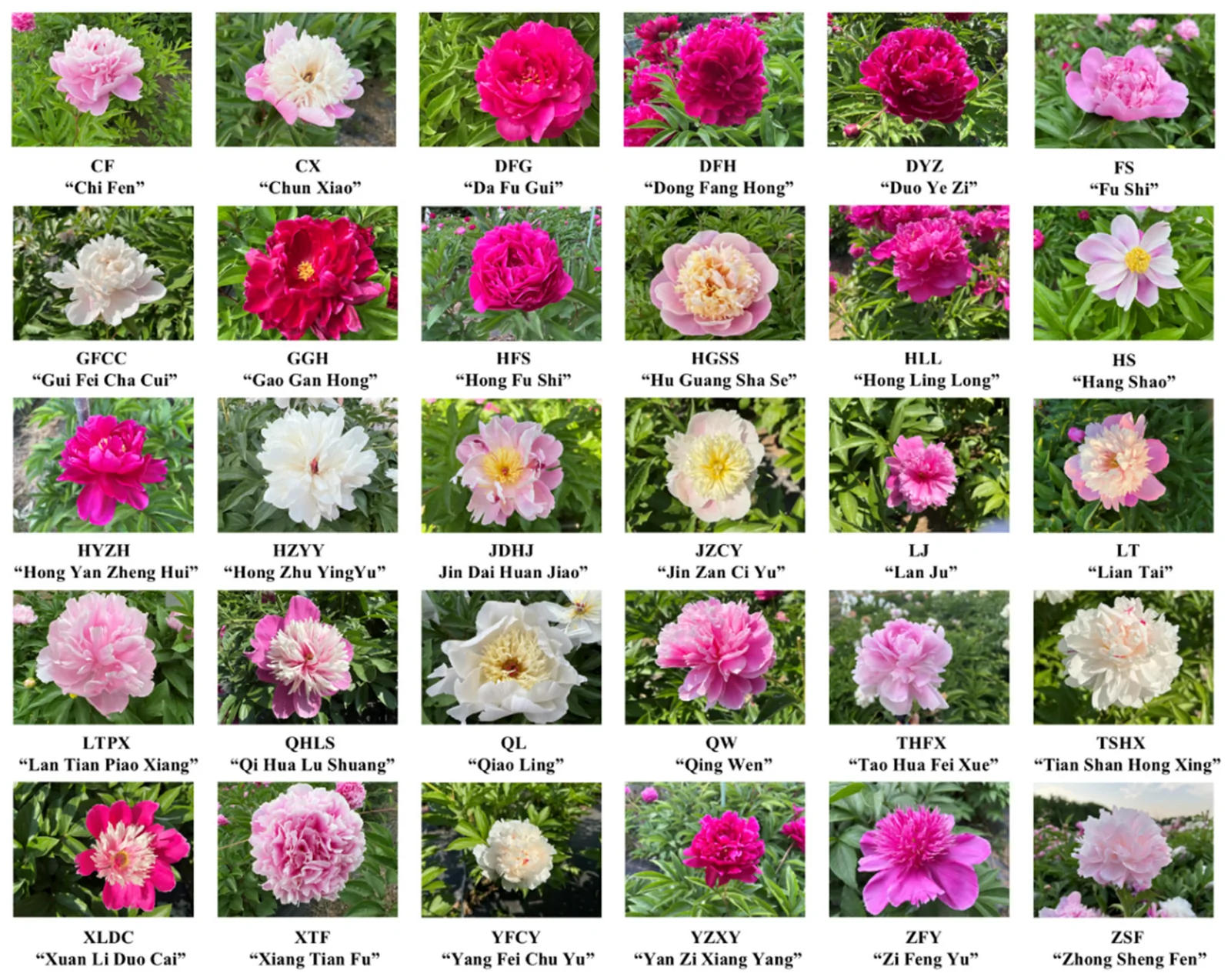
Photographs of the 30 *P. lactiflora* cultivars used in this study.

Plant materials were obtained from the Peony Resource Garden located at the Xiaotangshan Base of the National Flower Engineering Technology Research Center in Beijing, China (40°15′02″ N, 116°44′27″ E; altitude 31.6 m). A total of 30 ornamental cultivars belonging to the Chinese *P. lactiflora* cultivar group (LG) were chosen for floral volatile profiling (Figure 1). To ensure clarity, we have provided a complete abbreviation-to-name cross-reference table (for all 30 cultivars) in the Supplementary Materials (Table S3), and directed readers to this table in the caption of Figure 1. 

### 2.2. Instruments

Volatile analysis was conducted on a TurboMatrix 350 fully automatic thermal desorption system interfaced with a Clarus 690 gas chromatograph and a Clarus SQ 8T mass spectrometer (PerkinElmer, Waltham, MA, USA), with data acquisition and processing managed by TurboMass software (version 6.1.2). Trapping of volatiles was performed using CAMSCO stainless-steel (CAMSCO, Houston, TX, USA) thermal desorption tubes (1/4 inch × 3.5 inches, o.d. × length) packed with Tenax GR (60/80 mesh). Air sampling was carried out with a QC-1S atmospheric sampler (Beijing Institute of Labor Protection Science, Beijing, China).

### 2.3. Collection of Floral Volatile Compounds

Sampling was undertaken in May 2023, specifically on clear, windless mornings between 07:00 and 09:00. Plants at the same flowering stage were selected, and any dust or honeydew present on the sepals was gently wiped off prior to sampling.

For each cultivar, five flowers were enclosed together in a 5-L PVF sampling bag (Dalian Delin Gas Packaging Co., Ltd., Dalian, China). The bag was initially flushed with purified air using the atmospheric sampler equipped with color-changing silica gel, filling it to approximately 80% of its total volume. Immediately after inflation, a thermal desorption tube was connected to the sampler inlet to trap the headspace volatiles within this closed system. For each cultivar, five independent biological replicates were conducted. Each replicate consisted of five flowers sampled from different individual plants of the same cultivar. This experimental design was intended to capture biological variation.

Collection proceeded for 1 h at a constant airflow rate of 0.5 L min^−1^. Once sampling was complete, the adsorption tubes were promptly removed, capped, and stored under sealed conditions until GC–MS analysis.

### 2.4. Automated Thermal Desorption–GC–MS Analysis

Volatiles retained on the adsorption tubes were subjected to analysis using a fully automated two-stage thermal desorption process. Primary desorption was performed at 260 °C for 10 min with the cold trap maintained at −30 °C, followed by secondary desorption at 300 °C for 1 min, during which the cold trap was heated at a rate of 40 °C s^−1^.

The instrument was operated with the following gas flow parameters: outlet flow 20 mL min^−1^, carrier gas (high-purity helium) flow 1.5 mL min^−1^, desorption flow 20 mL min^−1^, and inlet flow 15 mL min^−1^. The injection split ratio was set at 4.0%.

Chromatographic separation was achieved on a DB-5MS capillary column (30 m × 0.25 mm i.d., 0.25 μm film thickness) (Agilent Technologies, Santa Clara, CA, USA). The oven temperature program was as follows: starting at 40 °C (held for 2 min), ramping to 180 °C at 6 °C min^−1^, then increasing to 270 °C at 20 °C min^−1^ and holding for 3 min.

Mass spectrometric detection was carried out in electron ionization (EI) mode at 70 eV. The ion source and transfer line temperatures were set to 230 °C and 250 °C, respectively, with full-scan acquisition covering a mass range of *m*/*z* 29–600.

### 2.5. ROAV Analysis

To identify the key odor-active compounds, we applied the Relative Odor Activity Value (ROAV) method established by Liu et al. [23]. The compound making the greatest contribution to the overall aroma (i.e., the maximum ROAV, denoted as ROAVmax) was assigned a reference value of 100. The ROAV for any other component i was then calculated using Equation (1):

(1) $${\mathrm{ROAV}}_{i}=\frac{C_{i}}{C_{\max}}\times \frac{T_{\max}}{T_{i}}\times 100$$

where

ROAVi—relative odor activity value of component i;

Ci—relative content of component I (%);

Ti—odor threshold of component I (μg kg^−1^);

C_max_—relative content of the compound contributing most to the aroma (%);

T_max_—odor threshold of the compound contributing most to the aroma (μg kg^−1^).

Although the relative content (Ci, %) and odor threshold (Ti, μg kg^−1^) are expressed in different units, their ratio Ci/Ti is used as a concentration-to-threshold index. The normalized ratio—calculated relative to the compound with the highest value—is dimensionless and is expressed as: ROAV_i_ = ROAVmax/100.

### 2.6. Data Analysis

Mass spectral data were processed using TurboMass (version 6.1.2), and compound identities were assigned by matching against the NIST 17 mass spectral library. Relative quantification of each volatile was accomplished via the peak-area normalization method. Principal component analysis (PCA) was performed using Origin 2024 software. The 28 volatile compounds included in the PCA were selected based on two criteria: (1) detection in at least 50% of the cultivars and (2) a mean relative content > 0.1% across all samples. To statistically assess the clustering patterns identified by PCA, an analysis of similarities (ANOSIM) was performed using Bray–Curtis dissimilarities, with petal color treated as a categorical factor. The analysis was performed in PAST v3.0 using 999 permutations [24]. One-way ANOVA was performed to assess differences in compound contents among the 30 cultivars, and between-cultivar coefficients of variation (CV) were calculated to quantify the degree of among-cultivar variation. The detailed results are provided in Supplementary Table S6.

## 3. Results and Discussion

### 3.1. Types of Aroma Components in Peony Flowers

**Figure 2:**
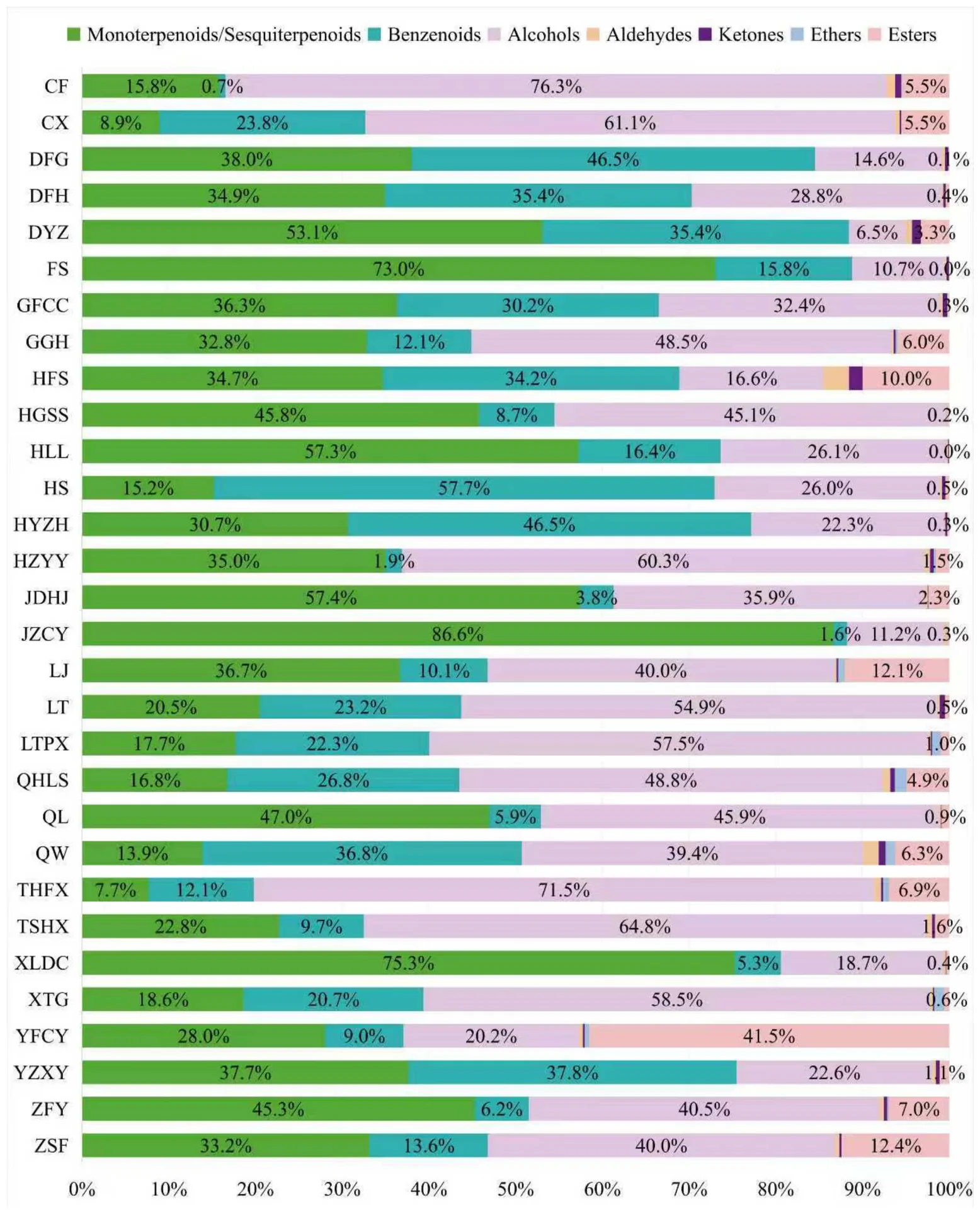
Relative contents of volatiles from fresh flowers of 30 peony varieties.

Across the 30 cultivars examined, seven chemical categories were detected among the floral volatiles: mono- and sesquiterpenes, benzenoid aromatics, alcohols, aldehydes, ketones, ethers, and esters. The inter-cultivar variations in the proportional abundance of these compound groups are visualized in Figure 2.

Monoterpenes and sesquiterpenes were the dominant compounds in several cultivars. Their relative contents were particularly high in ‘Golden Hairpin Thorn Jade’, ‘Colorful’, and ‘Fuji’, reaching 86.62%, 75.27%, and 73.00%, respectively. In contrast, their proportions were much lower in ‘Chunxiao’ and ‘Peach Blossom Flying Snow’, accounting for only 8.90% and 7.67%.

Aromatic (benzene-ring) compounds also represented an important component of peony floral scent. Their relative contents were relatively high in ‘Hangshao’, ‘Da Fugui’, ‘Hongyan Zhenghui’, and ‘Yanzi Xiangyang’, reaching 57.73%, 46.52%, 46.47%, and 37.80%, respectively. By contrast, these compounds were present at very low levels in ‘Red Pearl Reflecting Jade’, ‘Golden Hairpin Thorn Jade’, and ‘Chi Fen’, accounting for only 1.90%, 1.58%, and 0.70%.

Alcohols were another major group of volatile compounds in several cultivars. The highest proportions were observed in ‘Chi Fen’, ‘Peach Blossom Flying Snow’, ‘Tianshan Red Star’, ‘Chunxiao’, and ‘Hong Zhu Ying Yu’, with relative contents of 76.27%, 71.55%, 64.83%, 61.07%, and 60.28%, respectively. In contrast, alcohol compounds were much less abundant in ‘Fuji’, ‘Jinzhanciyu’, and ‘Duoyezi’, where their proportions were only 11.16%, 10.70%, and 6.51%.

The content of ester compounds varied substantially among cultivars. ‘Yang Fei Goes Bathing’ showed the highest ester proportion, reaching 41.51%, whereas ‘Da Fugui’, ‘Hong Linglong’, and ‘Fuji’ contained only trace amounts (0.11%, 0.04%, and 0.03%, respectively).

Compared with other cultivars, ‘Red Fuji’ exhibited the highest proportions of aldehydes and ketones, accounting for 2.93% and 1.55%, respectively. Ether compounds were detected in relatively low amounts across all cultivars, with slightly higher levels in ‘Xiangtian Ge’ and ‘Lantian Fragrant’, accounting for 1.18% and 1.03%, respectively. Collectively, these findings confirm that *P. lactiflora* cultivars exhibit widely divergent volatile emission patterns, reflecting substantial chemotypic heterogeneity within the tested germplasm.

### 3.2. Floral Aroma Components and Their Relative Contents

The composition of floral volatile compounds differed markedly among *P. lactiflora* cultivars. Among the detected compounds, terpenes, aromatic (benzene-ring) compounds, and alcohols showed relatively high emission levels and constituted the major classes of floral volatiles. The identified aroma components and their relative contents in the 30 peony cultivars are summarized in Supplementary Table S1.

A total of 24 monoterpenes and sesquiterpenes were identified, representing the largest group of volatile compounds in both diversity and abundance. Common compounds included α-pinene, laurene, α-terpinene, D-limonene, β-terpinene, γ-terpinene, α-caryophyllene, and β-caryophyllene. Among these, rolen was the dominant component in ‘Golden Hairpin Thorn Jade’, ‘Colorful’, ‘Golden Belt and Ring Jiao’, and ‘Purple Phoenix Feather’, accounting for 65.51%, 47.95%, 38.99%, and 32.38%, respectively. β-Caryophyllene was the major compound in ‘Multi Leaf Purple’ and ‘Fuji’, with relative contents of 42.33% and 36.27%, respectively.

Seven aromatic (benzene-ring) compounds were identified, including benzyl ether, *p*-phenyldimethyl ether, and phenylethanol as common components. The content of *p*-phenyldimethyl ether was particularly high in ‘Hangshao’, reaching 42.48%. Phenylethanol showed the highest relative content in ‘Qingwen’ and ‘Da Fugui’, accounting for 36.19% and 32.57%, respectively.

Alcohol compounds also represented an important group of floral volatiles, with 15 compounds identified. Common alcohols included n-pentanol, leaf alcohol, n-hexanol, citronellol, methyl heptenol, eucalyptol, linalool, and nerol. Citronellol was the dominant compound in 11 cultivars, including ‘Chi Fen’, ‘Hong Zhu Ying Yu’, ‘Tianshan Red Star’, ‘Chunxiao’, ‘Gao Gan Hong’, ‘Peach Blossom Flying Snow’, ‘Xiangtian Ge’, ‘Lantian Fragrant’, ‘Liantai’, ‘Zhongsheng Fen’, and ‘Lan Ju’, with relative contents exceeding 30%. Eucalyptol showed the highest levels in ‘Qiaoling’ and ‘Lake and Mountain Scenery’, reaching 42.39% and 25.91%, respectively. Nerolidol was the major compound in ‘Qihua Dew Frost’ (43.98%), whereas linalool was the dominant compound in ‘Dongfanghong’ (26.64%).

‘Yang Fei Goes Bathing’ contained relatively high levels of ester compounds, with ethyl acetate as the dominant component (35.11%). In contrast, aldehydes, ketones, ethers, and esters were generally present at low levels in most cultivars. Among these compounds, decanal, hexanal, and hexyl acetate were commonly detected fatty-derived volatiles.

Our dataset reveals that the floral volatile profiles of the 30 *P. lactiflora* cultivars differed substantially—not only in constituent identities but also in their proportional abundances—underscoring the considerable fragrance diversity present in this germplasm.

In this study, based on Supplementary Table S4, one-way ANOVA was performed to assess differences in compound contents among the 30 peony cultivars, and the between-cultivar coefficient of variation (CV) was calculated to quantify the degree of variation among cultivars. Supplementary Table S6 shows that all 61 compounds differed significantly among the 30 peony cultivars (*p* < 0.05), with 98.4% of the compounds showing highly significant differences (*p* < 0.001). The average between-cultivar CV was 119.74%, indicating a high degree of heterogeneity in compound contents among cultivars. Between-cultivar variation was on average 9.23 times greater than within-cultivar variation, providing strong evidence that cultivar effects were substantially greater than random variation.

### 3.3. ROAV Analysis of Volatile Components in Peony Flowers

Peony fragrance perception depends jointly on volatile concentrations and their corresponding odor detection thresholds. To assess the relative importance of each volatile constituent to the overall scent, we employed the Relative Odor Activity Value (ROAV) approach. Within this framework, higher ROAVs translate to greater contributions to the aroma profile.

The ROAV framework assigns compounds scoring ≥ 1.00 to the category of key odorants, whereas those in the 0.10–1.00 range are regarded as aroma-modifying agents that contribute to the overall fragrance complexity. Compounds with ROAV < 0.10 are regarded as potential aroma components with minor contributions. The odor threshold values reported in the literature were used to calculate ROAVs of floral volatiles from the 30 *P. lactiflora* cultivars according to Equation (1). The calculated ROAVs are presented in Supplementary Table S2.

A total of 10 common key aroma compounds with ROAV > 10 were identified in peony flowers, including ocimene, β-terpinene, terpinolene, *p*-phenyldimethyl ether, phenethyl alcohol, 1-pentanol, eucalyptol, linalool, citronellol, and cis-3-hexenyl acetate. These compounds play a dominant role in shaping the characteristic fragrance of peony flowers. Overall, peony floral scent is characterized mainly by rose aroma and citrus aromas, accompanied by sweet, woody, grassy, and slightly medicinal notes.

In addition, several compounds with 0.10 ≤ ROAV < 10 also contribute significantly to the fragrance profile. These include terpene compounds such as laurene, α-terpinene, D-limonene, and β-caryophyllene; alcohol compounds such as leaf alcohol, n-hexanol, n-nonanol, and nerol; and aldehydes and esters such as hexanal, citral, and hexyl acetate. Although their ROAVs are lower than those of the main aroma compounds, they play important roles in modifying and enriching the characteristic aroma of peony flowers.

Furthermore, some compounds were detected only in certain cultivars, including methyl cinnamate, nerol, and citronellyl acetate. These compounds contribute fruity, sweet, rose aroma, and orange-blossom-like aromas, thereby enhancing the diversity of fragrance characteristics among peony cultivars.

Overall, the ROAV analysis revealed that multiple volatile compounds jointly contribute to the formation of peony floral scent, resulting in diverse aroma profiles among different cultivars.

### 3.4. Principal Component Analysis of Characteristic Aroma Compounds in Peony Flowers

To explore the interrelationships among peony cultivars and their volatile constituents, and to pinpoint the principal determinants of floral scent, we carried out PCA using Origin 2024 on a dataset comprising 28 major volatiles detected across the samples. The 28 compounds selected for PCA all showed significant cultivar effects (*p* < 0.05; Supplementary Table S6), supporting their inclusion in the ordination analysis.

This analysis yielded four principal components having eigenvalues exceeding 1.0 (Supplementary Table S7), which together explained 71.89% of the total variance. This cumulative contribution rate suggests that the extracted components capture the majority of the information embedded in the original volatile profiles and adequately represent the key fragrance-related variation among cultivars.

Therefore, the first four principal components were considered sufficient to represent the major fragrance characteristics of peony flowers and were used for subsequent analysis of aroma profiles among cultivars.

Each loading value corresponds to the correlation coefficient between a given volatile compound and a given principal component. The magnitude of this value (i.e., its absolute term) reflects the relative importance of that compound in defining the component, with higher absolute loadings signifying greater influence. A loading with a positive sign denotes a direct correlation with the component, whereas a negative sign denotes an inverse one. Table S7 lists the loadings of the 28 selected volatiles across the four principal components. Varimax rotation was evaluated and yielded clustering patterns similar to those obtained from the unrotated PCA. Therefore, the unrotated PCA results are presented.

For the first principal component (PC1), 19 volatile compounds, including β-caryophyllene, n-hexanal, α-caryophyllene, and n-hexanol, showed positive loadings, indicating that these compounds contributed positively to PC1. In contrast, nine compounds, including nerol, citronellyl acetate, citral, and geranyl acetate, showed negative loadings and therefore contributed negatively to PC1.

For the second principal component (PC2), 17 compounds such as n-nonanol, citronellol, citral, and m-cymene exhibited positive loadings, whereas 11 compounds, including linalool, methyl cinnamate, ocimene, and cis-3-hexenyl acetate, showed negative loadings.

For the third principal component (PC3), 11 volatile compounds, including citral, eucalyptol, laurene, and n-nonanol, contributed positively, while 17 compounds, such as dimethyl ether and *m*-cymene, showed negative contributions.

For the fourth principal component (PC4), 16 volatile compounds, including linalool and citronellol, showed positive loadings, whereas 12 compounds, including decanal, geranyl acetone, and n-nonanol, exhibited negative loadings.

**Figure 3:**
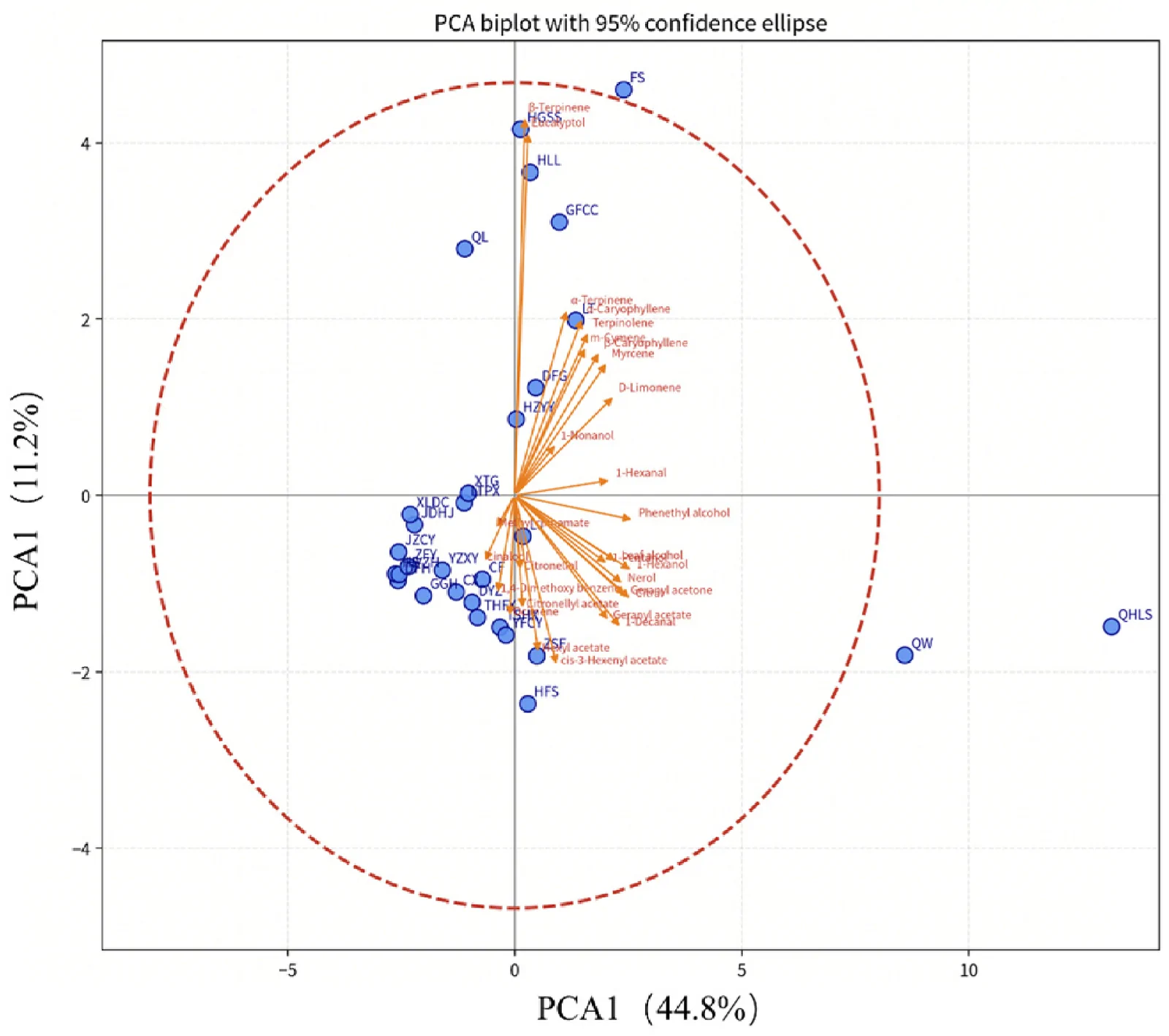
PCA biplot for 28 characteristic aroma components.

**Figure 4:**
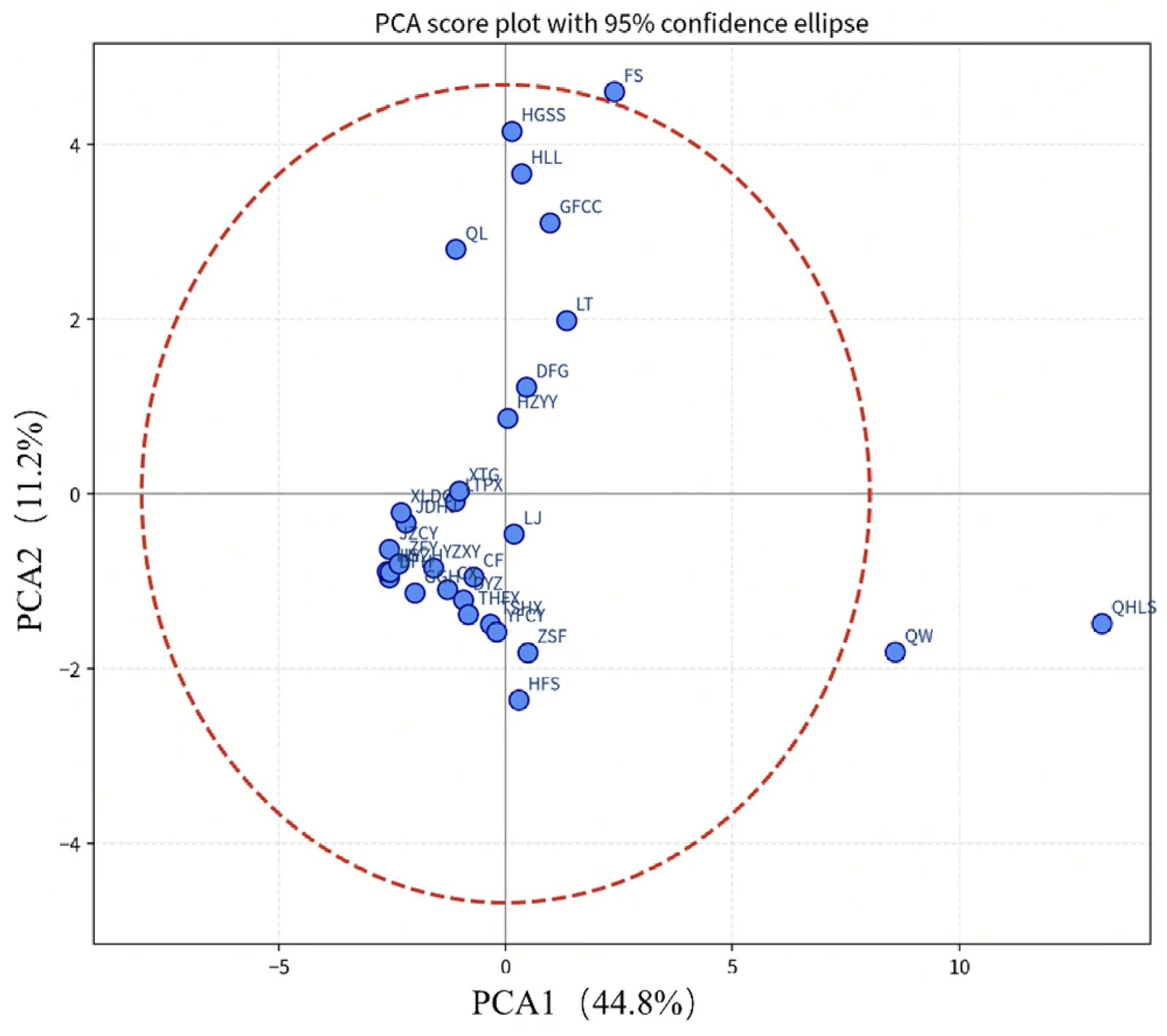
PCA biplot for 30 peony varieties.

To further visualize the relationships among cultivars and volatile compounds, a three-dimensional scatter plot was constructed using PC1, PC2, and PC3 as the X-, Y-, and Z-axes, respectively. The distribution patterns of the 28 key aroma compounds and the 30 peony cultivars are shown in Figure 3 and Figure 4. It should be noted that a cultivar’s position in the PCA score plot represents its overall volatile profile relative to the loading structure of the dataset. Thus, a negative loading for a compound on a given PC does not indicate that the compound is absent from a cultivar; rather, it indicates an inverse association with the compounds contributing positively to that axis.

Combining the spatial distribution of cultivars with the volatile compound profiles revealed that peony cultivars with similar flower colors tended to cluster together and exhibited similar aroma characteristics.

White-flowered cultivars, including ‘Golden Hairpin Thorn Jade’, ‘Yang Fei Goes Bathing’, ‘Guifei Chaqing’, ‘Tianshan Red Star’, ‘Red Pearl Reflecting Jade’, and ‘Qiaoling’, were located close to each other in the PCA space. Their fragrance profiles were mainly influenced by compounds such as ocimene, β-terpinene, and citronellol, resulting in a sweet floral aroma accompanied by fresh fruity notes.

Purple-red cultivars, such as ‘Yanzi Xiangyang’, ‘Dongfanghong’, and ‘Hongyan Zhenghui’, were also clustered together. Their floral scent was primarily associated with basilene, *p*-phenyldimethyl ether, linalool, and acetate compounds, producing sweet floral, herbal, and woody fragrance characteristics.

Pink-flowered cultivars, including ‘Xiangtian Ge’, ‘Lantian Fragrant’, and ‘Lan Ju’, were mainly influenced by compounds such as rose ether, citronellol, citral, and nerol. These compounds showed relatively high emission levels in these cultivars and possessed low odor thresholds (0.005–0.08 mg kg^−1^). Consequently, these varieties exhibited a fragrance profile characterized by a sweet rose aroma with fresh grassy and fruity notes. The ANOSIM results (R = 0.45, *p* = 0.018 < 0.05) confirmed that the volatile profiles differed significantly among petal color groups, supporting the PCA-based clustering observations.

Overall, cultivars with lighter flower colors, such as white, pink, and rose-red varieties, tended to cluster together in the PCA space. However, their aroma profiles were influenced by multiple volatile compounds rather than by a single dominant component, indicating that the fragrance characteristics of peony flowers result from the combined effects of multiple volatile substances.

## 4. Conclusions

### 4.1. Major Chemical Composition of Fresh Peony Flower Aroma

Seven classes of compounds were identified across the 30 cultivars: monoterpenes and sesquiterpenes, benzenoids, alcohols, aldehydes, ketones, ethers, and esters. Terpenoids, benzenoids, and alcohols collectively accounted for over 80% of the relative content in most cultivars, forming the primary matrix of peony floral scent. This finding aligns with previous reports that terpenoids and alcohols dominate the aroma profile of herbaceous peonies [25]. However, relative contents of different compound classes varied considerably among cultivars, indicating distinct chemotype differentiation. For instance, cultivars such as ‘Jinzanciyu’, ‘Xuanli Duocai’, and ‘Fuji’ were dominated by monoterpenes and sesquiterpenes (>73%), whereas ‘Chifen’, ‘Taohua Feixue’, and ‘Chunxiao’ were dominated by alcohols (>60%). Cultivars like ‘Hangshao’ and ‘Dafugui’ contained high proportions of benzenoids, while ‘Yangfei Chuyu’ was notable for its ester content (41.514%). Such compositional differences provide a direct chemical basis for the classification of peony fragrance types. It should be noted that volatile collections were performed during a single flowering season (May 2023); therefore, the potential effects of seasonal variation, phenological stage, and day-to-day weather conditions cannot be excluded. Future studies involving multi-year sampling across multiple phenological stages will be necessary to validate and refine the fragrance typology proposed here.

### 4.2. Key Aroma Components and Their Contributions to Fragrance Characteristics

The contribution of a volatile compound to overall aroma depends not only on its concentration but also on its odor threshold. ROAV analysis revealed that ten compounds—ocimene, β-terpinene, terpinolene, 1,4-dimethoxybenzene, phenethyl alcohol, 1-pentanol, eucalyptol, linalool, citronellol, and cis-3-hexenyl acetate—exhibited ROAVs greater than 10 in the majority of cultivars and were thus identified as core aroma contributors. Together, these compounds impart a complex fragrance dominated by rosy and citrus notes, accompanied by sweet, woody, and green nuances, along with a characteristic herbal-spicy undertone typical of peonies.

The key aroma-active compounds varied markedly among cultivars, directly shaping their distinctive fragrance profiles. In ‘Jinzanciyu’ and ‘Xuanli Duocai’, ocimene (ROAV = 100) contributed prominent herbal and sweet floral notes. Cultivars such as ‘Chifen’, ‘Hongzhu Yingyu’, and ‘Tianshan Hongxing’ were dominated by citronellol (ROAV = 100), yielding an intense rose-like scent. ‘Dongfanghong’ and ‘Yanzi Xiangyang’ were characterized by linalool (ROAV = 100), which imparts a strong sweet-woody green note. In ‘Hangshao’ and ‘Hongyan Zhenghui’, 1,4-dimethoxybenzene (ROAV = 100) provided sweet and woody tonalities. Additionally, compounds such as myrcene, D-limonene, β-caryophyllene, leaf alcohol, hexanal, and hexyl acetate, with ROAVs between 0.1 and 10, played important roles in modifying and harmonizing the overall fragrance profile. These results indicate that evaluating aroma contributions solely on the basis of relative content may be misleading; ROAV analysis, which incorporates odor thresholds, provides a more accurate reflection of the sensory weighting of individual components.

### 4.3. Relationships Between Aroma Composition and Flower Color

PCA extracted four principal components with eigenvalues greater than 1, accounting for 71.8% of the cumulative variance and representing the major variation in peony floral scent. Examination of the loading matrix showed that PC1 correlated positively with terpenoids and aliphatic compounds such as β-caryophyllene, hexanal, and α-caryophyllene, and negatively with compounds like nerol and citronellyl acetate. This pattern may reflect a metabolic trade-off between “terpenoid/woody-green” and “ester/alcohol-sweet” biosynthetic pathways.

In the three-dimensional PCA score plot, cultivars with similar flower colors tended to cluster spatially, suggesting an association between aroma composition and petal pigmentation. White-flowered cultivars (e.g., ‘Jinzanciyu’, ‘Yangfei Chuyu’, ‘Qiaoling’) were influenced mainly by ocimene, β-terpinene, and citronellol, resulting in sweet floral and fresh fruity scents. Purple-red cultivars (e.g., ‘Duoyezi’, ‘Dongfanghong’) were closely associated with ocimene, 1,4-dimethoxybenzene, and linalool, exhibiting herbal and woody characteristics. Certain pink-flowered cultivars (‘Xiangtiange’, ‘Lantian Piaoxiang’, ‘Taohua Feixue’) showed relatively high emissions of rose oxide, citronellol, citral, and nerol. Given the low odor thresholds of these compounds, their combined presence gave rise to a predominantly sweet rose fragrance with fresh green and fruity undertones. However, some light-colored cultivars did not show clear clustering based on aroma components, indicating that flower color alone is insufficient to fully predict fragrance type and that multiple factors likely regulate scent formation.

## Data Availability

Data will be made available on request.

## Supplementary Materials

The following supporting information can be downloaded at: https://www.mdpi.com/article/10.3390/plants15213490/s1, Supplementary Table S1. Aroma components and relative contents from fresh flowers of 30 peony varieties. Supplementary Table S2. Characteristic aroma components and corresponding ROAV values of fresh flowers of 30 peony varieties. Supplementary Table S3. Correspondence between abbreviated codes and Pinyin names of the 30 *Paeonia lactiflora* cultivars. Supplementary Table S4. Aroma components and relative contents from fresh flowers of 30 peony varieties-Replicate-level relative contents (n = 5). Supplementary Table S5. Characteristic aroma components and corresponding ROAV values of fresh flowers of 30 peony varieties-Replicate-level relative contents (n = 5). Supplementary Table S6. One-way ANOVA Results of Aroma Components Across 30 Peony Varieties (n = 5 replicates). Supplementary Table S7. Loadings matrix of 4 principal components.
